# Emerging scientists in analytical sciences: Sam Wouters

**DOI:** 10.1002/ansa.202200032

**Published:** 2022-08-29

**Authors:** Sam Wouters

**Affiliations:** ^1^ Janssen R&D Drug Metabolism and Pharmacokinetics Beerse Belgium


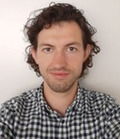
 In a series of editorials, Analytical Science Advances will highlight emerging scientists that work in the field of Analytical Sciences. For this first editorial, we have invited Dr. Sam Wouters to contribute to this Q/A. We are keen for anyone working in this field to nominate somebody for a Q&A by sending an email to one of the editors and explaining to us why this person should be highlighted.

Sam Wouters joined the Department of Chemical Engineering of the Vrije University Brussels (Brussels, Belgium) in 2012 as an MSc student designing and characterizing novel polymeric stationary phases for liquid chromatography. Later he continued as a PhD student in this department working on the miniaturization of chromatographic systems. Sam's motto is ‘*working hard and playing hard*’, which in fact means he is conducting cutting‐edge research with passion and lots of effort, which has led to an outstanding publication record, while outside work he also enjoys his time organizing great parties, making beautiful travels, etc. After his PhD, he joined Agilent Technologies, an instrument manufacturer that is the frontrunner of many analytical technologies. Only recently he moved back to Belgium and joined the R&D team Janssen Pharmaceutical developing novel analytical workflows for the characterization of emerging (bio‐)pharmaceuticals.

1

**How did you get involved in the field of analytical sciences?**

I was not a particularly good student at primary school, but when I started in trajectory called Technical Sciences in Belgium when I was 13, things got more interesting I guess. Chemistry, biology, physics: that seemed to make more sense to me. During a 2nd Master, I was combining polymer science with analytical chemistry, studying the synthesis of polymer monoliths to be used as stationary‐phase materials in liquid chromatography. This made me divert from material science into analytical science. Drawn to this field, the following 4 years I combined engineering and analytical chemistry, where the passion of Prof. Eeltink for the latter was certainly inspirational.

2

**What was the topic of your phd studies?**

The main idea was to miniaturize an entire ion chromatography (IC) system on a microfluidic platform, with the aim to minimize band‐broadening by integrating all components, and eventually realize a portable system. As this is of course a big endeavor, I focused on the key elements, which are the separation and detection. A first important step was to miniaturize the column. A lot of effort went into the in‐house manufacturing polymer chips containing a micromachined microfluidic channel. Optimization of the production process and the development of special metal encasing allowed us to establish a pressure rating up to 400 bar, which is more than suitable to do IC, where larger particles are still common use. The microchannels were packed with existing stationary phase materials. Moreover, I developed an approach to create macroporous interconnected polymer (monolithic) stationary phases in situ in the microchannels. Latex coating of the charged monolithic surface, with nanobeads of opposite charge, allowed me to establish the desired ion‐exchange retention and capacity. Another key component in IC is a suppressor, which is used to selectively remove ions from the eluent, making the system compatible with conductivity detection. I constructed a miniaturized chemically regenerated membrane suppressor, replacing sodium ions for protons, based on concentration gradient and diffusion. The protons and the sodium hydroxide in the eluent form water. In this way, full conversion of up to 80 mM NaOH to water was achieved applying flow rates as high as 20 µl/min, being compatible with the flow rates for the miniaturized column format. Moreover, a flow through ring‐electrode detector cell, yielding sub‐ppb level limits of detection, was developed, which was integrated at the outlet of the suppressor chip, located below the separation chip. Proof‐of‐concept was demonstrated with the analysis of minute amount of samples obtained from a 250‐year‐old Antarctic ice core.


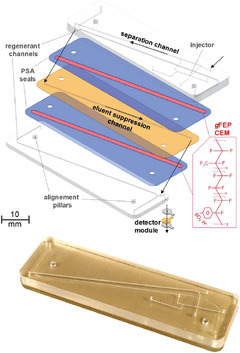


3

**What was your biggest achievement during this time?**

Despite having a challenging and multidisciplinary topic, I believe we got pretty far in developing components, which actually functioned. Next to successfully conducting my core research, at the same time I managed to contribute to the research of other people, which seemed fruitful with an additional 13 papers as a result. I was also able to personally present my own work during six oral lectures at international conferences, which gave me a great opportunity to connect with my peers and establish scientific collaborations.

4

**What advice would you give to recent phd graduates?**

I had the opportunity to learn from and work together with many very talented PhD students and post‐docs during my time, as well as renowned researchers from academia and industry such as Prof. Paul Haddad and Chris Pohl, which really lifted the research to a higher level. This opened a lot of doors, so my advice is to actively try to engage with such people at conferences, although I admit it can be a bit scary to approach ‘gurus’. Despite the above promotion of collaborations, it can also be a pitfall, and in some cases a reason why I see a lot of people struggling to in the end finalize their own PhD. For me it was important to stay focused on getting my own work done and at a certain point in time to prioritize this.

5

**Why did you to decide to join industry after your PHD study?**

For me, halfway through my PhD it was already clear to me I wanted to go into industry to generate more impact and work on solving relevant issues. There's so much interesting research happening at the universities, but often it ends up in a drawer after the graduation. After a short post‐doc period in industry, I joined the R&D team of Agilent Technologies in Germany. The idea that I was part of a team developing new technology in close collaboration with some big pharma companies really made this a very fulfilling job. At the same time, it still stayed a bit distant: I only helped provide others the tools to make the difference. I have now recently joined Jansen R&D to fulfil that desire to join a team, which makes the difference by bringing products to the market that will help people.

6

**Which skills were lacking for your position in industry and what changes would you suggest for the academic curriculum?**

Here, it is probably good to start with the quote of J.R. Oppenheimer, with which I opened my PhD booklet: ‘*No man should escape our universities without knowing how little he knows*’. No course can really prepare you for what is coming, as that will also vary greatly on the directions you will follow. There's no one‐fits‐all solution there, but I do think when it comes down to doing a PhD, what you will end up learning depends on your own drive and the development opportunities your promotor and you can generate. At a certain level, to many people in industry, the PhD shows a certain minimum qualification, but then the question will be: what else can you do? Knowledge is important but being able to convey knowledge and work together with respect bring you a long way.

7

**Can you describe current trends in analytical instrumentation for liquid chromatography?**

Having joined a research laboratory after my time at an instrument company, it has really sunk in: *I often have only 1 shot at getting the data*. Luckily vendors are aware of this, and they are following the trend towards more intelligence, with the aim of having more simplicity (but not per se below the hood of the system), and eventually the aim of reaching the goal of ‘zero‐failed analysis’. Examples would be automatic checks of the system suitability and performance, automatic start‐up, intelligent solvent‐level detection, etc. Apart from that there is also the need to solve complex analytical questions, which often involves using complicated setups, such as 2D‐LC. I do believe there has been much progress, in making this much more user‐friendly (usually by improving the software), and thus more accessible to a broader public.

Another example of really creating enabling technology would be the development of online LC, where the aim is to periodically sample directly from a reactor and have feedback loops in place to control the process. In this field of process analytical technologies, it is again software, which is critical to make the difference between being a nice tool, which can occasionally be used by experts, or something which can really be implemented in industry. The scheduling tool provided with the special sampler of the Agilent 1290 Infinity II Online LC allows to monitor processes easily, be it a 20‐min small molecule synthesis reaction, or a 3‐week fermentation in a bioreactor where multiple‐attributes have to be monitored.

8

**Which modern technologies are you currently establishing in your laboratory?**

I was provided the chance to move into the Pharma industry and join the Drug Metabolism and Pharmacokinetics group at Janssen R&D in Belgium, joining the team of Dr. Filip Cuyckens. As a scientist biotransformations the aim is to study the metabolites circulating in the body, to elucidate their structure using high‐resolution mass spectrometry and to establish a metabolic pathway. Eventually this contributes to the reports submitted to the Food and Drug Administration (FDA). Part of my job is also renewing existing technology and introducing novel instrumentation to expand on our capabilities. An important tool for our group is a setup capable of injecting very high volumes of sample, for example, 16 ml of plasma (with 50% organic) onto an liquid chromatographic system by making use of a trapping column. This is then transferred to a system with coupled columns where we do online radio‐activity detection and mass spectrometry, or fraction collection in 384 well plates with solid scintillation to perform offline counting with long counting times to detect low amount of radioactivity. Given the trend to dose with less radioactivity, the challenge is to inject more or improve detection limits. To better deal with very polar compounds, solvent effects and matrix effects, the capabilities of the setup will be expanded with at‐column dilution, which for example works very well with urine. Our group is non‐GxP, which means we have a lot of freedom in our experiments. Doing 4D‐LC for peak isolation would be a good example. More fundamental research also takes place; our laboratory for example has an ion trap with an infrared laser to study the spectrum of isolated molecules to aid in structure elucidation. An industry first.

9

**Where do you see yourself in 10 years?**

This is the kind of research group and field where there's so much to do and to learn, and it is not impossible to find me in this laboratory still. Of course, the work will not be the same anymore, as there's always the drive to invest in our analytical capabilities, and at the same time the organizations are changing into being more multi‐modality, meaning we will be solving more large‐molecules‐related questions, next to the small‐molecules drugs, which still remain important too.

10

**Can you say something about your hobbies outside the laboratory?**

Work‐life balance is obviously important to be on top of your game when at work. As such I like to clear the mind while climbing, it is just you and the rock. I'm mostly interested in traditional climbing, which is a discipline where you basically place all your protection yourself; hence you are fully focused and immersed into it. The technical aspects of that style of climbing make it particularly interesting to me. Next to sporty stuff, I like to study and collect WW1 artefacts.
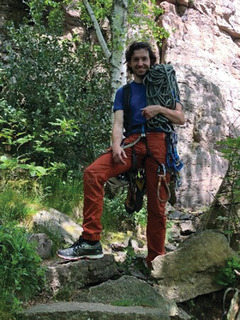


## CONFLICT OF INTEREST

The author declares that there is no conflict of interest that could be perceived as prejudicing the impartiality of the research reported.

